# Exporting Proteins Associated with Senescence Repair via Extracellular Vesicles May Be Associated with Early Pregnancy Loss

**DOI:** 10.3390/cells11182772

**Published:** 2022-09-06

**Authors:** Yi Zhang, Yunhui Tang, Xinyi Sun, Matt Kang, Min Zhao, Jiayi Wan, Qi Chen

**Affiliations:** 1 Department of Obstetrics & Gynaecology, The University of Auckland, Auckland 1141, New Zealand; 2 Department of Family Planning, The Hospital of Obstetrics & Gynaecology, Fudan University, Shanghai 200082, China; 3 Department of Gynaecological Cancer, Wuxi Maternity and Child Health Hospital Affiliated Nanjing Medical University, Wuxi 214002, China; 4 Department of Pathology, Wuxi No 2 People’s Hospital, Nanjing Medical University, Wuxi 214002, China

**Keywords:** placental EVs, proteomics, sorting of cargo, inhibitor of EV formation, missed miscarriage, senescence-repair, placental development

## Abstract

Introduction: Dysfunction of placental development is involved in early pregnancy loss. Senescent changes have been seen in missed miscarriage, one type of pregnancy loss. Extracellular vesicles (EVs) have been widely implicated in the pathogenesis of diseases. In this study, we investigated the protein profiles in placental EVs derived from missed miscarriage in comparison with healthy pregnancy. We also investigated whether cargos packed into EVs are involved in the dysfunctional development of the placenta seen in missed miscarriage. Methods: Proteomic analysis of placental EVs derived from healthy and missed-miscarriage placentae was performed. Three senescence-repair-associated proteins, replication protein A-70 (RPA-70), proteasome activator subunit-4 (PMSE-4), and protein activated kinase-2, (PAK-2) were examined in placental EVs and placentae, and in placental explants that had been treated with or without GW4869, by western blotting and immunohistochemistry. Results: The total number of proteins associated with placental EVs was not different between the two groups. However, there were 106 and 151 abundantly expressed proteins associated with placental micro- or nano-EVs from missed miscarriage in comparison with EVs from controls. Of these abundant proteins, 59 and 81 proteins in placental micro- or nano-EVs, respectively, are associated with DNA damage/repair and cell death/survival. We further found higher levels of three senescence-repair-associated proteins (RPA-70, PMSE-4, and PAK-2) associated with placental EVs, but lower levels of these proteins in missed-miscarriage placentae. Regarding inhibition of EV formation or release by GW4869, we found that the expression of these three proteins was higher in GW4869-treated placental explants from missed miscarriage. Discussion: Our data may suggest that “inadvertently” sorting of cargos and exporting proteins associated with senescence-repair by placental EVs may be associated with the dysfunction of placental development seen in missed miscarriage.

## 1. Introduction

Early pregnancy loss during the first trimester is the most common complication of early pregnancy before 20 weeks and affects 10% to 20% of all pregnancies [1]. Although chromosomal abnormalities or endocrinological disorders contribute to half of early pregnancy losses [2], dysfunction of placental development, including morphological and functional changes in placental trophoblast cells, is associated with many complicated pregnancies, including miscarriage [3,4,5]. Placental development is regulated by the balance of trophoblast proliferation and apoptosis in the development of the placenta in normal pregnancy [6]. Disbalance in placental trophoblast apoptosis is associated with abnormal pregnancy outcomes including miscarriage [7,8,9].

The normal trophoblast life cycle involves the proliferation and differentiation of mononucleated cytotrophoblasts, some of the cells then merge into the overlaying multinucleated syncytiotrophoblasts [10]. Turnover of aged syncytiotrophoblasts is thought to involve apoptosis, followed by shedding into the maternal circulation as extracellular vesicles (EVs) [11], from as early as six weeks of gestation [12]. EVs, including placental EVs, are lipid-enclosed particles that are released from all cells studied to date, including the placental syncytiotrophoblast, and carry many functional proteins, regulatory RNAs, DNA, and lipids (reviewed in [13]). EVs play an important role in cell-to-cell communication, including signaling during pregnancy, which has roles in maternal vascular and immune adaptation [14,15]. A number of studies have shown that placental EVs derived from complicated pregnancy impact the function of target cells [13] by releasing cargos that EVs carry, such as dangerous proteins including misfolded proteins and Mixed Lineage Kinase domain-Like (MLKL) [16].

One of the formations of EV biogenesis, called exosomes, includes three processes: (1) the formation of inside endocytic vesicles, (2) the generation of multivesicular bodies, (3) the release of vesicles [17]. It is now well known that EVs contain diverse molecules, including various proteins [18,19] that are originally derived from the cell of origin [20]. Cargos are sorted by endosomal sorting complexes required for transport (ESCRT)-dependent or ESCRT-independent mechanisms [21]), and EV cargos are subject to change under stress conditions [22]. This may suggest that there is a possibility that during EV biogenesis, functional proteins or regulatory RNAs are “inadvertently” packed into EVs and released under pathological conditions. Oxidative stress may affect lipids of the exosome membrane and the RNA composition in the exosomes [23,24].

There is growing evidence that suggests that increased placental oxidative stress contributes to the common pathogenesis of early pregnancy loss which may result in impaired trophoblast invasion [5,25,26]. Increased oxidative stress can induce DNA damage in the placentae [27], and disrupt the protein-folding process and enhance the production of misfolded proteins [28]. An increased level of senescence is associated with complications of pregnancies, such as preeclampsia [27] and missed miscarriage [29]. We have recently reported that instead of being released by EVs, proteins that are associated with senescence, DNA damage, and endoplasmic reticulum (ER) stress are accumulated in missed-miscarriage placentae [29].

The differences in proteomic characterizations in placental EVs derived from missed-miscarriage and normal first-trimester placentae has not been investigated yet. Therefore, we undertook this study to investigate the proteomic profiles in placental EVs derived from missed miscarriage. We next analyzed the potential mechanisms of these proteins that are associated with cellular senescence in the pathogenesis of missed miscarriage, a subtype of early pregnancy loss. We further investigated whether there is an “inadvertent” exportation of functional proteins associated with senescence repair by EVs in missed miscarriage.

## 2. Materials and Methods

This study was approved by the ethics committee of The Hospital of Obstetrics & Gynaecology of Fudan University, Shanghai, China (reference number 201862), and Wuxi Maternity and Child Health Hospital affiliated with Nanjing Medical University, Wuxi China (reference number: 2021/01020204). All placentae were collected with an informed written patient consent form.

### 2.1. Placentae Collection and EVs Preparation

Proteomics analysis: missed-miscarriage placentae (*n* = 3) and gestation-matched first-trimester placentae from elective surgical termination (*n* = 3) were collected from the Family Planning Clinic in The Hospital of Obstetrics & Gynaecology of Fudan University. The gestational age for missed miscarriage ranged from 9 to 12 weeks.

Functional analysis: 14 placentae were collected from missed miscarriage and 14 healthy placentae were collected from elective surgical termination from Wuxi Maternity and Child Health Hospital affiliated with Nanjing Medical University, Wuxi, China.

Missed miscarriage is defined as where the fetus has died or not developed, without any maternal symptoms, and the fetus has not been physically miscarried. All the potential contributing factors with chronic diseases such as chronic hypertension, renal disease, and diabetes mellitus were excluded. All the placentae (missed miscarriage and healthy controls) were collected from surgical evacuation. Placental EVs were harvested from placental explant culture as described previously [30]. Briefly, after removal of the decidua, approximately 400 mg of first placental explants (wet weight) were dissected and were then cultured in Netwell™ culture inserts (400 µm mesh) at 37 °C in Advanced DMEM/F12 containing 2.5% FBS and 1% penicillin/streptomycin in an ambient oxygen atmosphere containing 5% CO_2_ overnight. The conditioned media were collected, and the cellular debris was removed by centrifuging at 2000× *g* for 10 min. The supernatant was centrifuged at 20,000× *g* for 1 h for micro-EV collection. The supernatant was further centrifuged at 100,000× *g* for 1 h for nano-EV collection (Avanti J30I Ultracentrifuge, JA 30.50 fixed angle rotor, Beckman Coulter).

In some experiments, the missed-miscarriage placental explants (*n* = 5) were cultured in the presence or absence of GW4869 (20 µM) (MedChemExpress, Shanghai, China) for 24 h. The placental explants were then fixed by 4% PFA for 24 h and paraffin blocks were prepared. GW4869 is a commonly used inhibitor of EV formation or release (reviewed in [31]).

Morphology of placental micro and nao-EVs was confirmed by electron microscopy and molecular characterization of CD81, an EV-enriched marker, and pan-cytokeratin, a marker of placental origin, was confirmed by western blotting (Supplementary Figures S1–S3).

### 2.2. Protein Extraction and Quantification

Proteins in placental micro- or nano-EVs derived from six placentae were extracted by commercially purchased RIPA buffer (Applygen Technologies Inc., Beijing, China). After centrifugation by 12,000 rpm for 15 min to remove the cellular debris, the proteins were collected and stored at −80 °C for future experiments. The protein concentrations of placental micro- or nano-EVs were measured by Bicinchoninic Acid (BCA) assay following the manufacturer’s guidelines (Beyontime Technology, Guangdong, China). The concentration was normalized to µg/mg placental tissue.

### 2.3. Proteomic Analysis

Proteomic analysis was performed with a Fusion mass spectrometer (ThermoFisher Scientific, Massachusetts, USA). The dried peptide fractions were applied to a C18 nanocapillary column (3 μm, 250 mm × 75 μm, Eksigent Technologies, Redwood City, CA, USA) and eluted into the Orbitrap Fusion LC-MS/MS mass spectrometer (ThermoFisher Scientific, Massachusetts, USA). Buffer A (0.1% formic acid in water) and buffer B (0.1% formic acid in acetonitrile) were used for gradient elution. Then, the peptides were eluted from the column at a constant flow rate of 600 nL/min (in total 4 µL) with a gradient of buffer B from 5% to 30% in 82 min. The eluted peptides were ionized in positive-ion mode at 2000 V and scanned in the Orbitrap with a resolution of 120,000 which covered the range 300–1400 m/z. Dynamic exclusion was used for the data collection with an exclusion duration of 18 s, and the minimum intensity was 5000. Proteomic analysis was performed in three individual samples and the data was pooled as a mean.

### 2.4. Data Analysis

Protein identification was performed using the MaxQuant (version 1.6.0.16, Jürgen Cox, Max Planck Institute of Biochemistry, Martinsried, Germany), searching against the Uniport database of human protein sequences (http://www.uniprot.org). Search parameters were of trypsin specificity. Variable modifications were defined as oxidation on methionine residues and acetylation of protein *n*-terminal, and carbamidomethylation on cysteine was the fixed modification. The resulting peptide groups only have peptides with modified sites, removing contaminant matches and matches to the reverse database. For the quantitation of proteins, metaboanalyst software (http://www.metaboanalyst.ca/) was used. Unpaired Student’s t-tests were used to compare the two groups. The significant differentially expressed proteins (DEP) (*p*-value < 0.05) were further selected and the ones with a differential expression ratio of over ±2 were obtained, referred to as abundantly expressed proteins. Upstream regulator analysis, functional analysis, downstream effect analysis and network between proteins in the DEP dataset were performed by ingenuity pathway analysis (IPA) software (http://www.ingenuity.com/products/ipa). For IPA analysis, Fisher’s exact test was used to analyze the significance of canonical pathways and the association between proteins and biofunctions. Z-score was used to determine the potential activation states (activated or inhibited) of implicated biological processes.

### 2.5. The Levels of Protein Activated Kinase-2 (PAK-2), Proteasome Activator Subunit-4 (PMSE-4) and Replication Protein A-70 (RPA-70) Carried by Placental EVs Were Measured by Western Blotting

After protein extraction from placental micro- or nano-EVs (*n* = 4), the proteins (20 µg) were denatured by boiling with loading buffer (Cwbio, Beijing, China), followed by electrophoresis on 10% SDS-PAGE gels for 1 h 30 min. Proteins were transferred onto PVDF membranes (0.22 μm, Biosharp Inc., Hefei, China) and were blocked with 5% non-fat milk for 1 h at room temperature. After incubation with primary antibodies at 4 °C overnight, the membranes were then washed with TBS-T and were cultured with a secondary antibody (Affinity, Shanghai, China, 1:5000) for 1 h at room temperature. The membranes were treated with ECL (Vazyme, Nanjing, China) and developed on the chemical fluorescence luminescence developer. The concentrations of senescence-repair-associated antibodies are as follows: PAK-2 (Abcam, Shanghai, China), 1:5000; PMSE-4 (Abcam, Shanghai, China), 1:1000; RPA-70 (Abcam, Shanghai, China), 1:1000.

Semiquantitative analysis of the western blotting images after normalization to levels of b-actin were performed by measuring the density of the band with ImageJ. Data show mean and standard deviation (SD).

### 2.6. The Expression of PAK-2, PMSE-4 and RPA-70 in Placentae Collected from Missed-Miscarriage or Healthy First-Trimester Placentae or Collected from Missed-Miscarriage Placental Explant Culture Was Measured by Immunohistochemistry

The expression of PAK-2, PMSE-4, and RPA-70 in placentae collected from missed miscarriage (*n* = 5) or control (*n* = 5) or from missed-miscarriage placental explant culture in the presence or absence of GW4869 (*n* = 5) was measured by immunohistochemistry. Paraffin blocks were sectioned with 5 µm and the expression of PAK-2, PMSE-4, or RPA-70 was examined. Briefly, after being deparaffinized in xylene and rehydrated in graded alcohol, the sections were boiled with citrate buffer (pH = 6.8) for 2 min using a pressure cooker for antigen retrieval. Rabbit anti-human PAK-2 (1:200, Abcam, Shanghai, China), rabbit anti-human PMSE-4 (1:1000, Abcam, Shanghai, China), or rabbit anti-human RPA-70 monoclonal antibody (1:100, Abcam, Shanghai, China) was then added to the sections for 1 h at room temperature. After washing with PBS-T three times, the sections were incubated with a secondary antibody and streptavidin/peroxidase complex using Vectastain Universal Quick Kit (VectorLabs, Newark, CA, USA) following the instructions. After further washing with PBS-T three times, sections were incubated with 3,3-Diaminobenzidine (DAB) for visualization. Sections were then counter-stained for 1 min with hematoxylin. Negative controls were performed as above but omitting the primary antibodies.

Semiquantitative analysis of the immunohistochemistry result was performed on the strength of staining, which was scored by two independent authors (QC and YZ). Strong staining was scored as 3 points, moderate staining was scored 2 points, and weak staining was scored as 1 point compared with the negative control, as we used previously [29]. Data show mean and standard deviation (SD).

### 2.7. Statistical Analysis

The protein contents of placental EVs were expressed as mean and standard deviation (SD). Student’s *t*-test was performed for the statistical analysis using Prison (version 9.4). Semiquantitative analysis of the western blots or the immunohistochemistry images was assessed by *t*-test (nonparametric) using the Prism software package; data were expressed as mean and SD. *p* < 0.05 was considered as statistically different.

## 3. Results

### 3.1. There Was No Difference in the Total Protein Quantity Carried by EVs Derived from Missed Miscarriage and Controls

**Figure 1:**
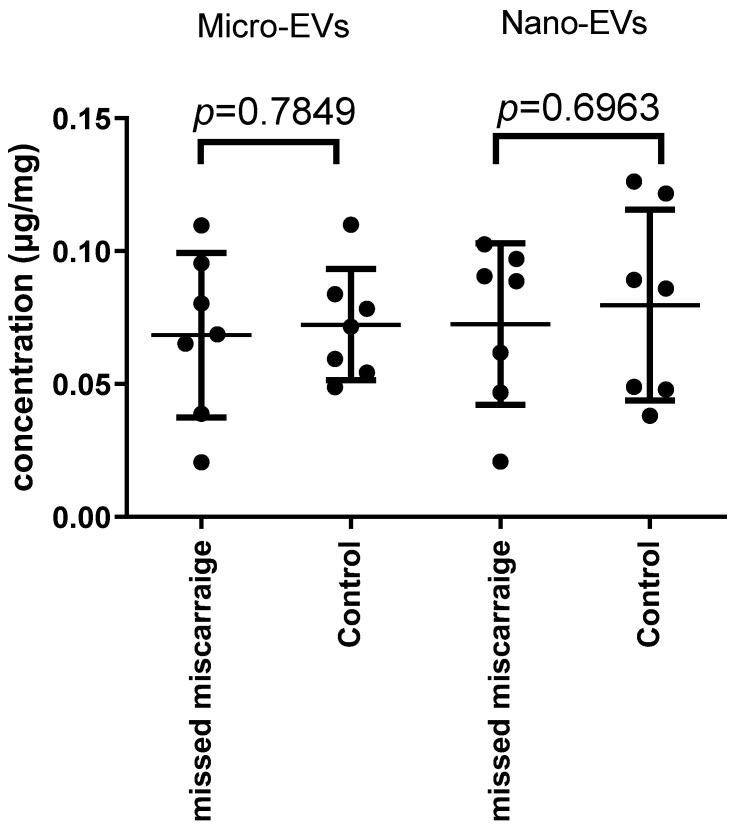
The concentration of proteins in micro-EVs and nano-EVs from missed miscarriage and healthy placentae.

We first compared the total protein quantity in placental EVs derived from control or missed-miscarriage placentae. There was no statistical difference in the protein concentration of EVs from healthy and missed-miscarriage placentae (Figure 1).

### 3.2. Comparison of the Number of Proteins Carried by Placental EVs Derived from Controls and Missed Miscarriage

**Figure 2:**
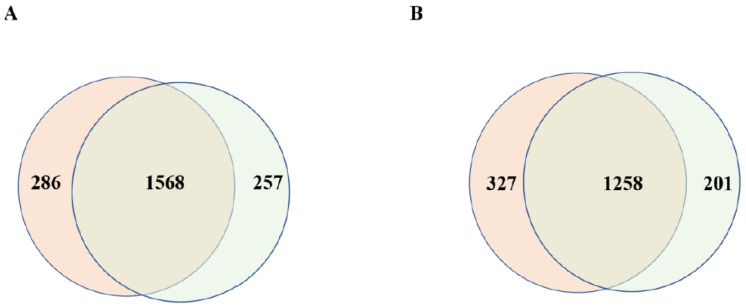
The number of proteins in placental micro-EVs (**A**) or nano-EVs (**B**) from missed miscarriage and healthy pregnancy.

**Figure 3:**
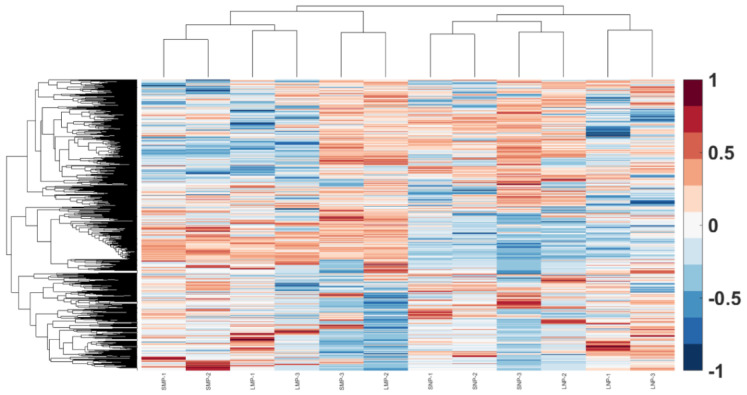
The heatmap of the proteins in placental EVs from missed miscarriage and healthy pregnancy.

To understand the difference in placental micro- or nano-EVs derived from controls and missed-miscarriage placentae at the proteomic level, we performed a proteomic analysis. We identified 1854 or 1825 proteins in placental micro-EVs derived from healthy or missed-miscarriage placentae, respectively. Of these, there were 1568 overlapping proteins between the two groups (Figure 2A). In addition, 1585 or 1459 proteins were identified in placental nano-EVs derived from the control or missed-miscarriage placentae, respectively. Of these, there were 1258 overlapping proteins between the two groups (Figure 2B). There was no difference in the number of proteins identified in placental EVs between the two groups. Furthermore, the number of overlapped proteins between placental micro- and nano-EVs from missed miscarriage was similar to the overlapped proteins between placental micro- and nano-EVs from controls. The expression levels of non-overlapped proteins between the two groups were much lower. Thus, further analysis was carried out on the overlapped proteins. The heatmap of the overlapping proteins is shown in Figure 3.

### 3.3. Analysis of Abundantly Expressed Proteins in EVs from Missed Miscarriage

**Figure 4:**
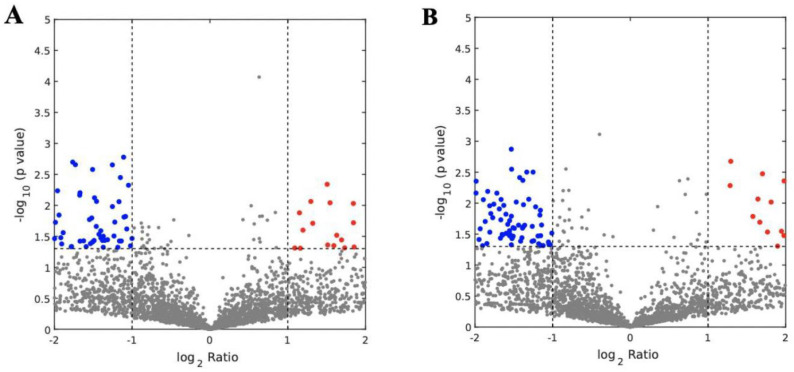
The abundantly expressed proteins in micro-EVs (**A**) and nano-EVs (**B**) from missed-miscarriage and healthy placentae.

By proteomic analysis, the expression of 106 proteins in placental micro-EVs were significantly different between the two groups. Among these, 37 proteins were upregulated, and 69 proteins were downregulated, compared with micro-EVs derived from the healthy first-trimester placentae (Figure 4A). In addition, the expression of 151 proteins in placental nano-EVs was significantly different between the two groups. Among these, 31 proteins were upregulated, and 120 proteins were downregulated, compared with nano-EVs derived from the healthy first-trimester placenta (Figure 4B). Detailed proteins that were abundantly expressed are summarized in Supplementary Tables S1 and S2.

### 3.4. Functional Analysis of Placental EVs

Following IPA functional analysis, we found that the abundantly expressed proteins associated with micro- or nano-EVs are mainly associated with biological functions, physiological system development and functions, and diseases and disorders (Supplementary Tables S3 and S4). There were 20 abundantly expressed proteins associated with DNA damage and repair, and 39 abundantly expressed proteins associated with cellular death and survival in placental micro-EVs, respectively. There were 15 abundantly expressed proteins associated with DNA damage and repair, and 66 abundantly expressed proteins associated with cellular death and survival in placental nano-EVs, respectively. Of these proteins, we selected three senescence-repair proteins, RPA-70 (Replication protein A 70) and PAK-2 (Protein Activated Kinase 2) which were highly expressed in placental micro-EVs, and PSME-4 (Proteasome Activator Subunit 4, also called PA 2000) which was highly expressed in placental nano-EVs, for further analysis. RPA-70 binds ssDNA to protect it and maintains it in an unfolded state. PSME-4 potentially regulates cellular homeostasis at the transcription level and regulates the expression of genes involved in cell survival. PAK-2 regulates cell motility, cell cycle progression, apoptosis, or proliferation. PAK-2 is an important regulator of cellular senescence.

**Figure 5:**
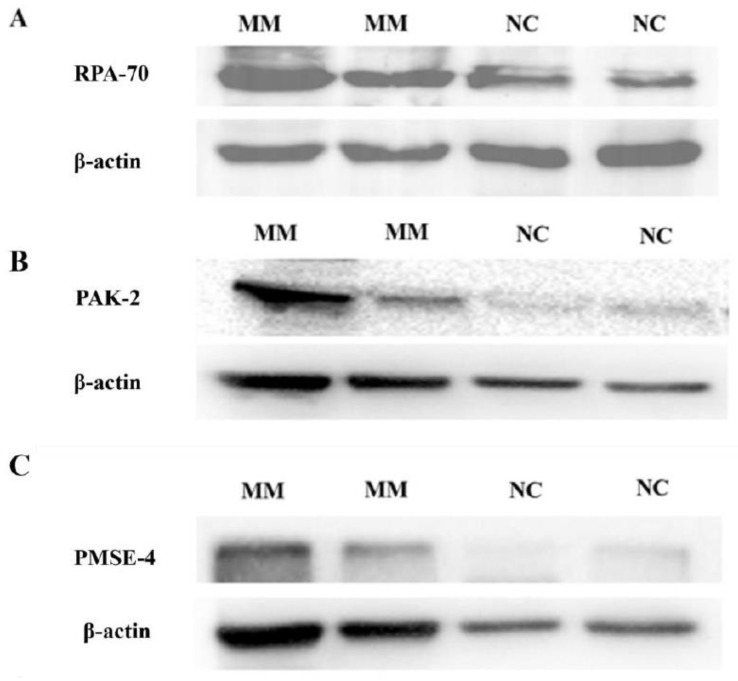

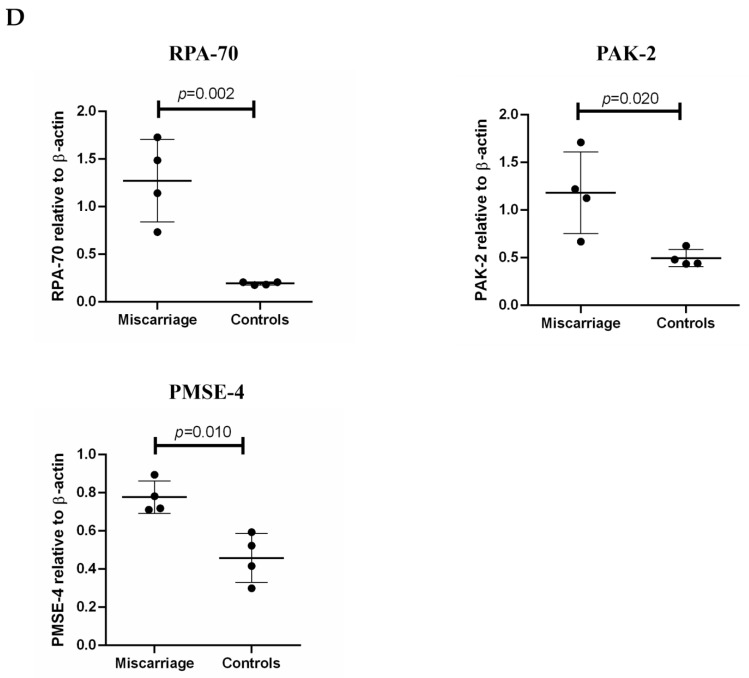
The representative western blotting images showing the high levels of RPA-70 (**A**) and PAK-2 (**B**) in placental micro-EVs, and high levels of PSME-4 (**C**) placental nano-EVs derived from missed-miscarriage placentae, measured by a semiquantitative analysis (**D**). (MM: missed miscarriage, NC: control).

**Figure 6:**
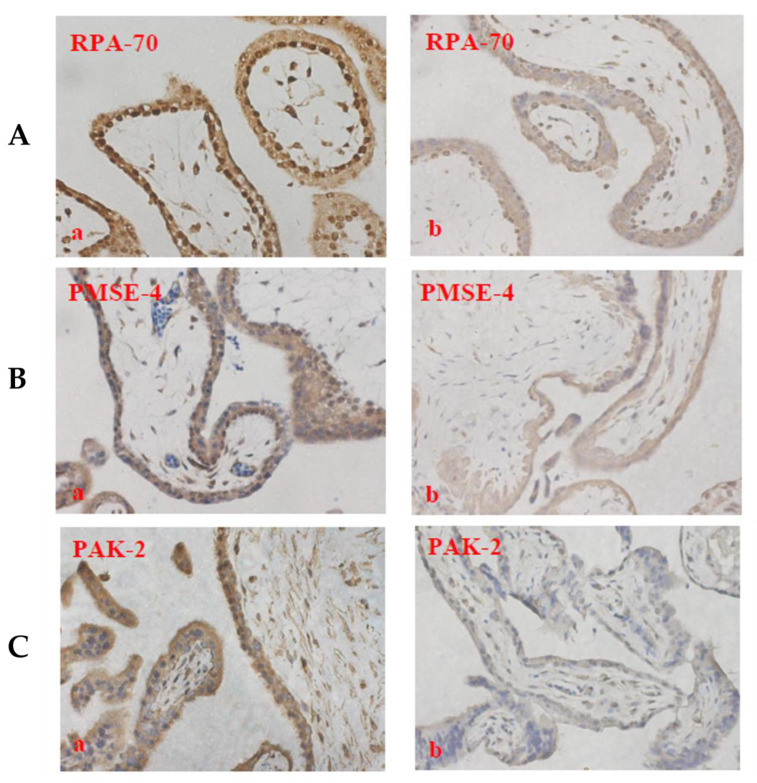

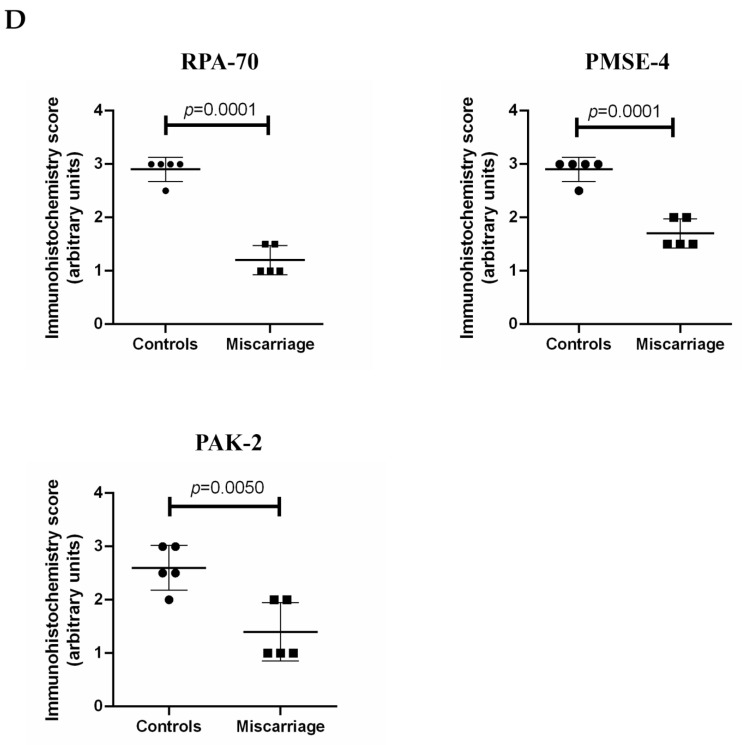
The representative immunohistochemistry images showing the lower expression of RPA-70 (**A**), PSME-4 (**B**), and PAK-2 (**C**) in missed-miscarriage placentae (**b**) and control placentae (**a**), measured by a semiquantitative analysis (**D**) (magnification: ×400).

**Figure 7:**
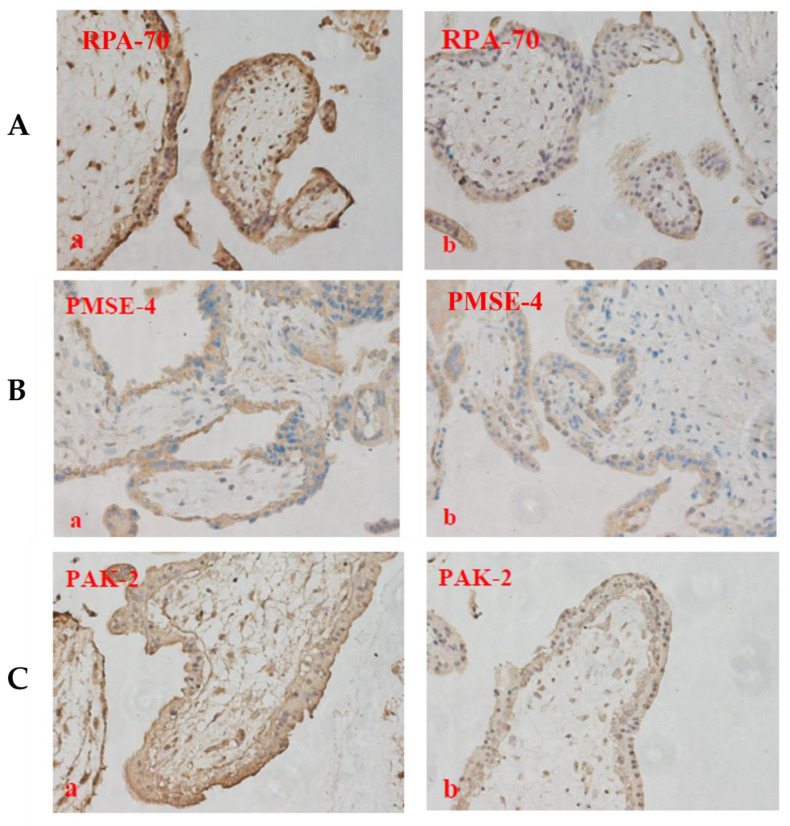

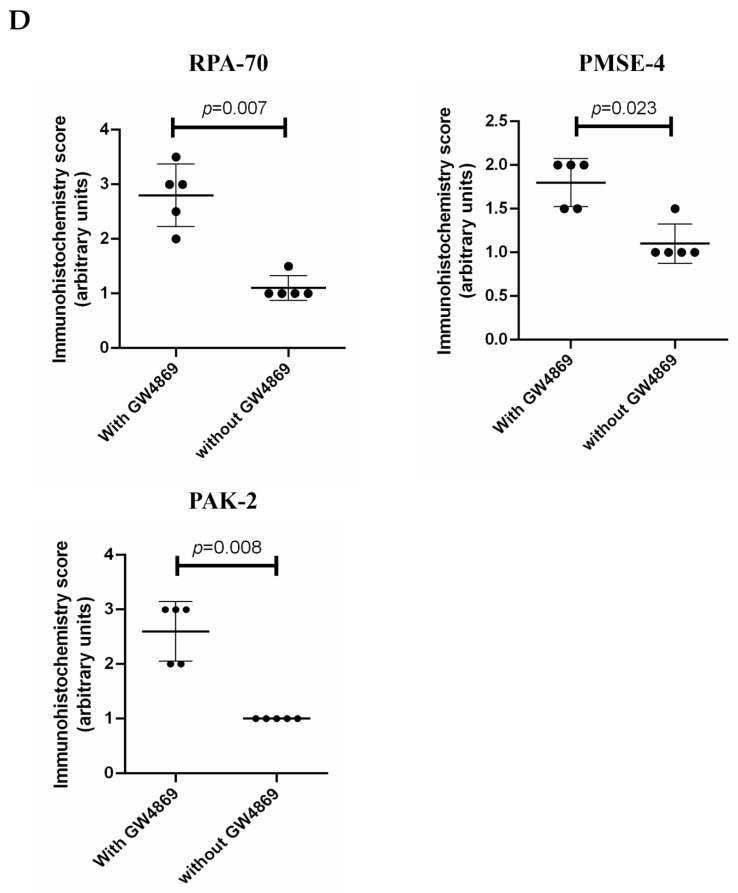
The representative immunohistochemistry images showing the expression of RPA-70 (**A**), PSME-4 (**B**), and PAK-2 (**C**) in missed-miscarriage placental explants that had been treated with (**a**) or without (**b**) GW4869, measured by a semiquantitative analysis (**D**) (magnification: ×400).

We first investigated the levels of these proteins in placental EVs from missed-miscarriage placentae, and the tissues from which the EVs were derived, since our recent study reported an accumulation of proteins associated with senescence (DNA damage) in missed-miscarriage placentae [29]. As shown in Figure 5, the levels of RPA-70 and PAK-2 in placental micro-EVs, or the levels of PSME-4 in placental nano-EVs derived from missed-miscarriage placentae were significantly increased, compared with controls. In contrast, the expression of RPA-70, PAK-2, and PSME-4 was significantly lower in the missed-miscarriage placentae, compared with their expressions in the healthy first-trimester placentae, measured by a semiquantitative analysis (Figure 6A–D). GW4869 is a commonly used inhibitor of EV formation or release (reviewed in [31]). We next investigated the expression of these proteins in missed-miscarriage placental explants that had been treated with GW4869. The expression of RPA-70, PAK-2, and PSME-4 in missed-miscarriage placental explants that had been treated with GW4869 were significantly higher than in untreated placental explants from missed miscarriage, measured by a semiquantitative analysis (Figure 7A–D). Interestingly, the expression of RPA-70, PAK-2, and PSME-4 in healthy first-trimester placental explants that had been treated with GW4869 was not different to that of the untreated placental explants (data not shown).

## 4. Discussion

In this study, we found no differences in the total protein quantity carried by placental EVs derived from missed miscarriage and healthy placentae. By proteomic analysis, we found no differences in the number of proteins associated with placental EVs derived from missed miscarriage and healthy placentae. However, there were 106 or 151 differently expressed proteins in placental micro- or nano-EVs between the two groups. Higher levels of cellular senescence-repair proteins, RPA-70, PSME-4, and PAK-2 were seen in placental EVs derived from missed miscarriage, but lower levels of the three proteins were seen in missed-miscarriage placentae, compared with healthy first-trimester placentae. Inhibition of EV formation or release by GW4869 resulted in higher levels of RPA-70, PSME-4, and PAK-2 in missed-miscarriage placental explants.

Extracellular vesicles are described as a heterogeneous population of membrane-bound vesicles released by all cell types studied to date, including both prokaryotic and eukaryotic cells, from unicellular to multicellular organisms. Therefore, the secretion of EVs is considered as an evolutionarily conserved process [32,33,34]. EV biogenesis involves double invagination of the plasma membrane and the formation of intracellular multivesicular bodies. The intracellular multivesicular bodies can then release intraluminal vesicles into the extracellular space upon fusion with the plasma membrane (reviewed in [24]), although the biogenesis of EVs depends on the subtypes of EV. It is well-recognized that cargos carried by EVs, including functional proteins and regulatory RNAs, are originally packed from cells [20], and the contents associated with EVs vary with the subtypes of EVs, cell type, and physiologic conditions (reviewed in [35]). The functions of EVs are also dependent on EV cargos [36].

The functions of EVs have been widely studied, including their involvement in disease pathogenesis, as well as their utilization as diagnostic biomarkers for diseases by exploiting the changes in EV cargo signatures. However, the comparison of the proteomics profiles between missed miscarriage and healthy pregnancy has not been investigated yet. In our current study, we found the total protein in placental EVs derived from healthy or missed-miscarriage placentae was not different. Interestingly, by proteomic analysis, the numbers of proteins in placental EVs were also not different between the two groups. Although the protein contents of EVs often reflect their cell of origin [20], given that there were no differences in the total protein contents, our data suggested that the mechanisms involved in the biogenesis and release of EVs may be similar between the two different conditions of pregnancy. Quantity may not be a suitable indicator that the same EV packaging mechanisms are operating in the two pregnancy conditions.

By proteomic analysis, however, we found a difference in the levels of 106 upregulated and 151 downregulated proteins, respectively, in placental micro- and nano-EVs between the two conditions of pregnancy. Through IPA functional analysis, we found that there are 93 and 129 abundantly expressed proteins in placental micro- and nano-EVs, respectively, associated with protein synthesis, DNA damage, and cell death/survival (Supplementary Tables S3 and S4). These data suggest that these abundantly expressed proteins play a critical role in the pathogenesis of missed miscarriage, as we recently reported that senescence and DNA damage were associated with placental dysfunction in missed miscarriage [29].

When we selected three senescence-repair-associated proteins for further investigation, we interestingly found high levels of RPA-70, PSME-4, and PAK-2 in placental EVs derived from missed-miscarriage placentae. In contrast, there were lower levels of the three proteins in missed-miscarriage placental tissues from which the EVs were derived. RPA-70 binds and stabilizes single-stranded DNA intermediates that form during DNA replication or upon DNA stress [37]. PMSE-4 potentially regulates the expression of genes involved in cell survival upon selective mitochondrial inhibition in neuroblastoma cells [38]. PAK-2 is required for the expression of genes involved in cellular senescence and regulates the deposition of newly synthesized H3.3 onto chromatin in senescent cells [39]. We do not know the exact reason why these three proteins are enriched in EVs compared with the cell or origin. However, it could point towards differences in the mechanisms of cargo packaging onto EVs from placentae complicated by missed miscarriage. The packaging of cargos onto EVs is based on endosomal sorting complexes required for transport (ESCRT)-dependent or ESCRT-independent mechanisms. Post-translational modifications have been shown to contribute as a signal for cargo transport into multivesicular bodies and demonstrated that the ESCRT machinery plays a crucial role in this pathway [40]. EV proteins modified by post-translational modifications can be directly loaded onto the EVs, and the specific post-translational modifications also can control the selective mechanisms of protein cargo sorting and promote some proteins to be enriched in EVs [21]. Oxidation and redox processes are thought to affect the packaging of EV cargos. Importantly, oxidation may also affect lipids of the EV membrane [23], and cellular stress could alter the composition of cell-derived EVs [22]. Oxidative stress has been shown to influence the RNA composition in EVs [23]. We previously showed that oxidative stress contributes to the changes of senescence in missed-miscarriage placentae [29]. Taken together, our current data may suggest that dysfunctional packaging of cargos that causes exportation of some proteins involved in senescence-repair by EVs may be a potential mechanism in missed miscarriage.

To investigate this hypothesis, placental explants from missed miscarriage were treated with GW4869, a commonly used inhibitor of EV formation and release (reviewed in [31]). Interestingly, we found that missed-miscarriage placental explants that had been treated with GW4869 showed high expressions of RPA-70, PSME-4, and PAK-2, compared with untreated miscarriage placental explants. In contrast, explants from healthy first-trimester placentae that had been treated with GW4869 showed no difference in the expressions of these proteins, compared with untreated healthy placental explants. Our recent study found that potentially dangerous misfolded proteins were not exported into EVs, and consequently accumulated in missed-miscarriage placentae [29]. Therefore, our current data may suggest an “inadvertent” exportation of functional proteins associated with senescence repair by EVs in missed miscarriage, a pathological condition of pregnancy. Future study to understand the mechanism is required.

In conclusion, our data demonstrated that the total protein quantity, including the number of proteins, was not different in placental EVs derived from healthy and missed-miscarriage placentae. However, by proteomic analysis, there were 106 and 151 abundantly expressed proteins in placental micro- and nano-EVs derived from missed-miscarriage placentae, in comparison with the controls. High levels of three senescence-repair-associated proteins were seen in EVs derived from missed-miscarriage placentae, but lower levels of these three proteins were seen in missed-miscarriage placentae. Inhibition of EV formation or release resulted in increased levels of these three proteins in missed-miscarriage placentae. Our data may suggest that “inadvertently” or dysfunctional packaging of cargos and exportation of proteins involved in senescence repair by EVs may be associated with the dysfunction of placental development seen in missed miscarriage. Future research is required to confirm our findings.

## Data Availability

The datasets used and/or analyzed during the current study are available from the corresponding author on reasonable request. The raw proteomic data included in this study has been uploaded on iProX (accession number: IPX0002999003); visit http://www.iprox.cn for a direct link to the raw data accessed on June 2022.

## Supplementary Materials

The following supporting information can be downloaded at: https://www.mdpi.com/article/10.3390/cells11182772/s1, Figure S1: The electron image of placental micro-EVs (A) and nano-EVs (B); Figure S2: The size of micro-EVs and nano-EVs is confirmed by Nanosight NS300 (Malvern Panalytical, Almelo, The Netherlands); Figure S3: The characterizations of placental micro-EVs and nano-EVs are also confirmed by measuring EV-specific surface proteins, CD81 and pan cytokeratin. No expression of vimentin in placental micro- and nano-EVs suggests the trophoblastic origin of vesicles obtained from cultured placental explants.
