# Supplementary material for: Exporting Proteins Associated with Senescence Repair via Extracellular Vesicles May Be Associated with Early Pregnancy Loss
Source: Cells. 2022 Sep 6;11(18):2772. doi: 10.3390/cells11182772 (PMC9496689; doi:10.3390/cells11182772)

Supplementary

Figure S1: The electron image of placental micro-EVs (A) and nano-EVs (B)

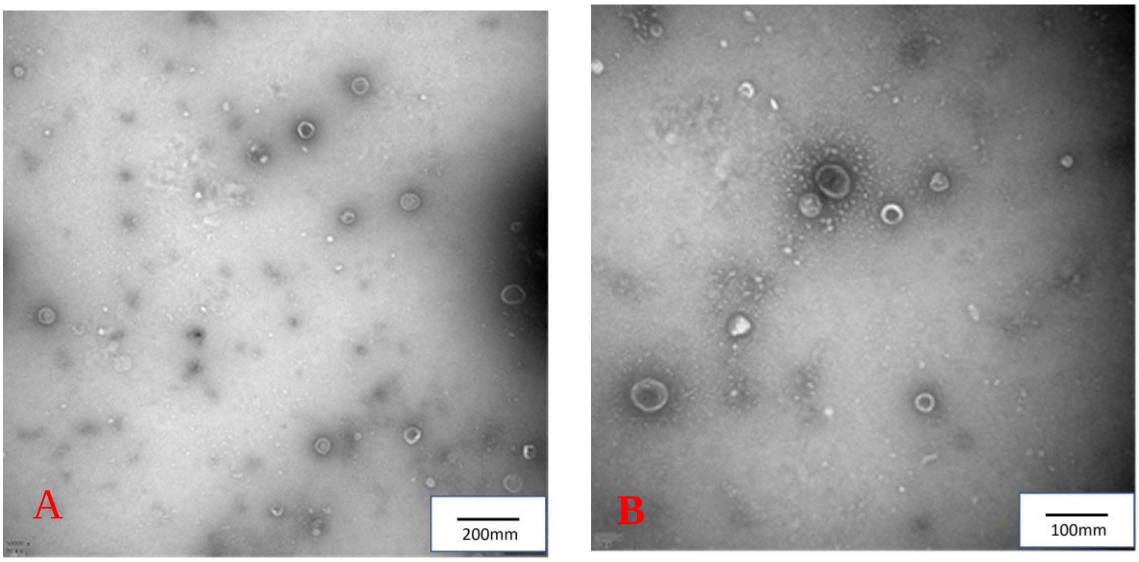

Figure S2: The size of micro-EVs and nano-EVs is confirmed by Nanosight NS300 (Malvern Panalytical, Almelo, The Netherlands).

Micro-EVs

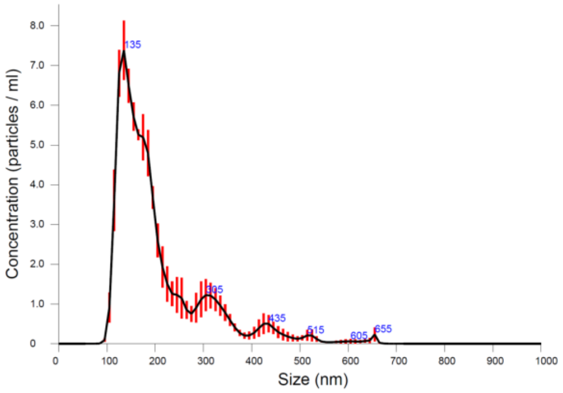

Nano-EVs

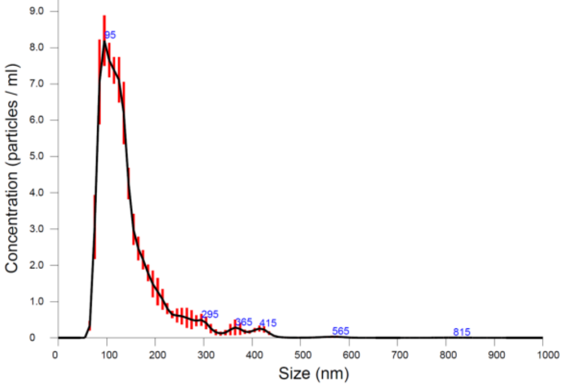

Figure S3: The characterisations of placental micro-EVs and nano-EVs are also confirmed by measuring EV specific surface proteins, CD81 and pan cytokeratin. No expression of vimentin in placental micro- and nano-EVs suggests the trophoblastic origin of vesicles obtained from cultured placental explants.

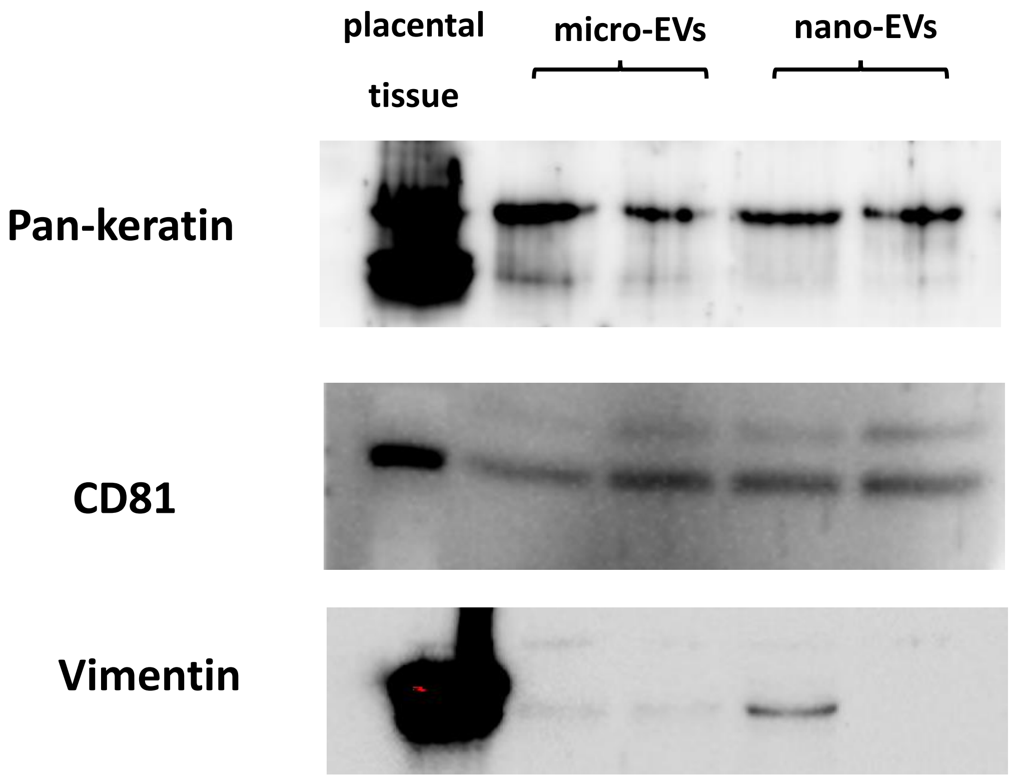

Supplement: Supplementary file 1 [file cells-11-02772-s001.zip › cells-1869949-supplementary.pdf]
